# Cardiac Autonomic Alteration and Metabolic Syndrome: An Ambulatory ECG-based Study in A General Population

**DOI:** 10.1038/srep44363

**Published:** 2017-03-14

**Authors:** Yan Ma, Ping-Huei Tseng, Andrew Ahn, Ming-Shiang Wu, Yi-Lwun Ho, Ming-Fong Chen, Chung-Kang Peng

**Affiliations:** 1 Division of Interdisciplinary Medicine and Biotechnology, Beth Israel Deaconess Medical Center, Harvard Medical School, Boston, MA, USA; 2 Department of Internal Medicine, National Taiwan University Hospital, Taipei, Taiwan; 3 Massachusetts General Hospital, Harvard Medical School, Boston, MA, USA

## Abstract

Metabolic syndrome (MetS) has been associated with chronic damage to the cardiovascular system. This study aimed to evaluate early stage cardiac autonomic dysfunction with electrocardiography (ECG)-based measures in MetS subjects. During 2012–2013, 175 subjects with MetS and 226 healthy controls underwent ECG recordings of at least 4 hours starting in the morning with ambulatory one-lead ECG monitors. MetS was diagnosed using the criteria defined in the Adult Treatment Panel III, with a modification of waist circumference for Asians. Conventional heart rate variability (HRV) analysis, and complexity index (CI_1–20_) calculated from 20 scales of entropy (multiscale entropy, MSE), were compared between subjects with MetS and controls. Compared with the healthy controls, subjects with MetS had significantly reduced HRV, including SDNN and pNN20 in time domain, VLF, LF and HF in frequency domain, as well as SD2 in Poincaré analysis. MetS subjects have significantly lower complexity index (CI_1–20_) than healthy subjects (1.69 ± 0.18 vs. 1.77 ± 0.12, p < 0.001). MetS severity was inversely associated with the CI_1–20_ (r = −0.27, p < 0.001). MetS is associated with significant alterations in heart rate dynamics, including HRV and complexity.

Metabolic syndrome (MetS), also known as the cardiometabolic syndrome, is a medical disorder that consists of a complex combination of abdominal obesity, hypertension, impaired glucose tolerance and dyslipidemia^1^,^2^. With those interrelated risk factors, MetS causes chronic damage to the cardiovascular system and thus is strongly linked with incident cardiovascular diseases (CVD), diabetes, and related mortality and morbidity. It is estimated that MetS affects 20–25% of adults in general population^3^. It has also been predicted that the incidence and prevalence of MetS will keep increasing^4^,^5^,^6^,^7^,^8^, imposing an inevitable and profound impact on global healthcare systems^9^. Compared with healthy population, people with MetS have a five-fold greater risk of developing type 2 diabetes^10^, twice as likely to develop CVD^11^, three times as likely to have a heart attack or stroke^9^,^12^. The more components of the MetS that are evident, the higher is the cardiovascular mortality rate^13^.

Reciprocal reinforcement of insulin resistance^14^,^15^,^16^ and sympathetic activity^17^,^18^,^19^ plays an important role in the pathophysiology of cardiac dysfunction. Cardiac autonomic function can be evaluated non-invasively with ECG-based measurements. Heart rate variability (HRV), including time domain, frequency domain and non-linear analysis, is one of the most frequently studied measurements with its predictive power^20^,^21^,^22^. In recent years, system complexity gradually becomes a more established theory to evaluate health. Decrease of complexity has shown to be a common sign of pathological conditions or aging^23^,^24^,^25^. Healthy physiologic function represents the body’s capacity to adapt to ever changing stresses by complex interactions between multiple control systems, feedback loops, and regulatory processes that operate over multiple scales of time and space^25^. Entropy measurement is considered an important index for complexity. Therefore, multiscale entropy (MSE)^26^,^27^,^28^ has been widely used to evaluate human health conditions on a system level, but has not yet been applied in subjects with MetS.

We hypothesized that subjects with MetS may have early cardiac autonomic dysfunction in terms of alterations of heart rate dynamics, which may further lead to significant cardiovascular comorbidities and complications. To test the hypothesis, we prospectively recruited subjects from a Chinese general population, who attended periodic health check-ups in our institute. All subjects have undergone a comprehensive evaluation for components of MetS, as well as an ambulatory ECG monitoring. We compared various ECG-derived measurements of cardiac autonomic functions between subjects with and without MetS. Furthermore, we tested the clinical utility of complexity index in the evaluation of the presence and the severity of MetS in the general population.

## Results

### Subjects and demographics

From Jan 2012 to June 2013, 175 subjects with MetS and 226 healthy subjects were identified and included in the final analysis (Table 1). Since age and gender were different in these two groups, comparisons of various outcome measures were adjusted for age and gender. Comparing with healthy subjects, subjects with MetS had significantly higher body mass index (BMI), waist circumference (WC), body fat percentage and blood pressure. In addition, more subjects with MetS had the comorbidities of sleep apnea. No significant difference was found in terms of mental status for the two groups. In terms of biometric parameters, significant difference presented on triglycerides, high density lipoprotein, both fasting and 2-hr postprandial blood glucose, hemoglobin A1c, high-sensitivity C-reactive protein and hemoglobin, while total cholesterol, low density lipoprotein and total protein showed no significant difference between groups.

### Time and frequency domain heart rate variability analyses

In time domain analysis of HRV (Table 2), subjects with MetS have faster heart rates and shorter heart beat intervals, after controlling for age and gender. Significant differences were also seen in SDNN and pNN20 before or after adjustment. No difference was seen in rMSSD and pNN50 even after adjustment. In frequency domain analysis (Table 2), decreased HRV was seen in MetS subjects, with significantly lower power in VLF, LF and HF bands. Poincaré analysis showed a significant difference in SD2 with or without adjustments. Further regression analysis adjusting for various confounding variables, including medication, mental status and sleep apnea, still confirmed the impact of MetS on various HRV parameters (Table 2).

### Multiscale entropy for complexity analysis

**Figure 1:**
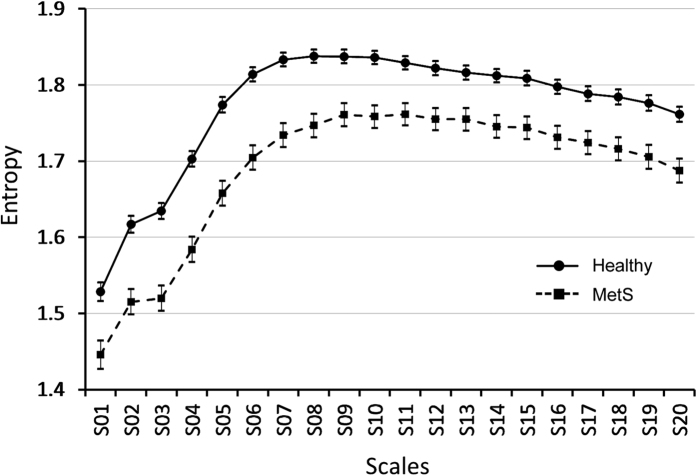
Comparison of MSE (CI_1–20_) between subjects with metabolic syndrome and healthy subjects. X axis: scale 1–20 of MSE. Y axis: the values of entropy. Data of the healthy and MetS groups are presented as mean ± standard error.

Complexity index (CI_1–20_) in MetS subjects was significantly lower than those in healthy subjects (1.69 ± 0.18 vs 1.77 ± 0.12, p < 0.001), and the difference remained significant even after adjustment for age and gender (Table 2). When investigated in different scales, MetS subjects had significantly lower entropies than healthy subjects in all scales (Fig. 1).

### MetS severity and heart rate dynamics

**Figure 2:**
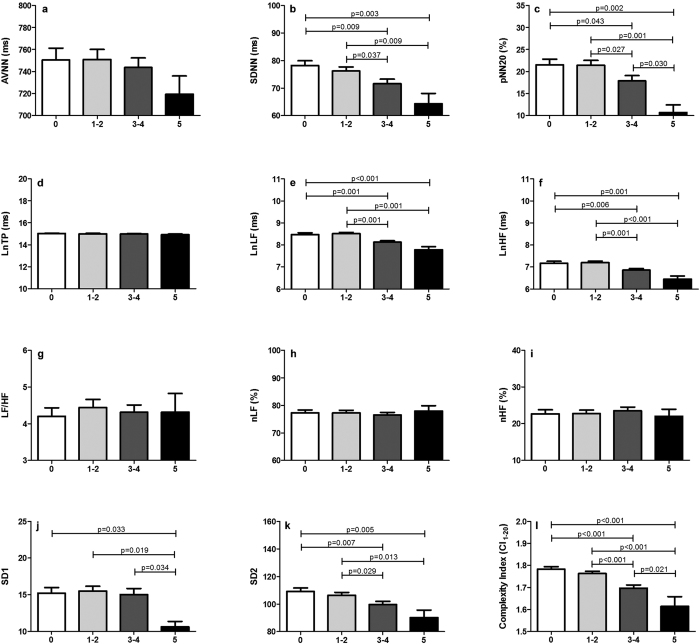
Heart rate dynamics and complexity for different MetS severity. AVNN, average of NN intervals; SDNN, the standard deviation of NN; pNN20, percentage of heart period differences >20 ms; LnTP, log form of total power; LnLF, log form of low frequency power; LnHF, log form of high frequency power; LF/HF, ratio of low frequency over high frequency; nLF, normalized low frequency power; nHF, normalized high frequency power; SD1, normalized deviation of instantaneous beat-to-beat N-N interval variability in the short diameter; SD2, normalized deviation of instantaneous beat-to-beat NN interval variability in the long diameter.

Following the definition of metabolic syndrome, all subjects were classified according to the numbers of metabolic derangement, as having 0, 1–2, 3–4, and 5 MetS components, which can be considered as a spectrum from healthy to severe MetS. As shown in Fig. 2, for daytime heart rate dynamics in time and frequency domain, mean NN, nLF, nHF and LF/HF did not show any difference with the severity of MetS, but SDNN and pNN20 showed significant difference in subjects with 3 or more MetS components. For SD1 in Poincaré analysis, only subjects with 5 MetS components showed significant difference, but not between other subgroups. SD2 is comparatively more sensitive than SD1. For complexity, subjects with 3 or more MetS components had significantly lower complexity index (CI_1–20_) than subjects with no or 1–2 MetS components.

### MetS severity and Complexity

**Figure 3:**
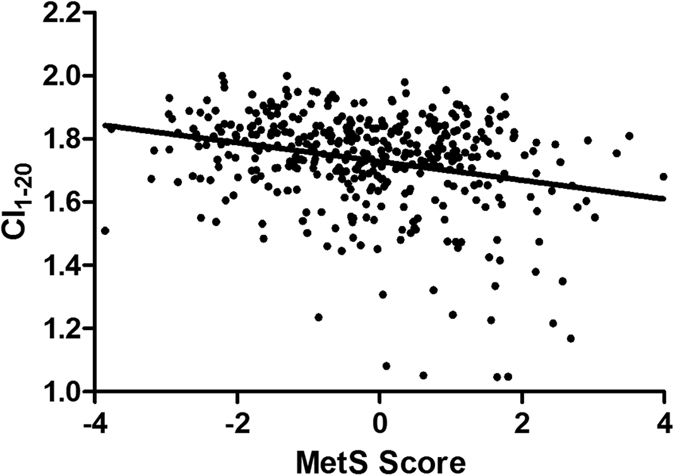
Correlation of metabolic syndrome severity, in terms of MetS Score, and complexity index (CI_1–20_). MetS Score = −11.8769 + (1.5432298 * Log Glucose) + (0.7872732 * Log Triglyceride) − (1.588791 * Log HDL) + (0.0277125 * WC) + (0.0232299 * SBP) + (0.0420722 * DBP) − (0.016408 * Age) − (0.73821 * Gender) Male = 1, Female = 0. p < 0.001.

As shown in Fig. 3, complexity index (CI_1–20_) was negatively correlated to MetS Score (Pearson’s correlation, r = −0.27, p < 0.001), suggesting that as the severity of metabolic derangement went up, the health level, as indicated by the complexity index, decreased.

## Discussion

This prospective study included healthy individuals and subjects with MetS from periodic health examinations and obtained comprehensive biometric measurements and metabolic profiles for advanced analysis. From daytime ECG characteristics, subjects with Mets had significantly reduced SDNN and pNN20 for HRV time domain, VLF, LF and HF in frequency domain, as well as SD2 in Poincaré analysis. Complexity index (CI_1–20_) in subjects with MetS was significantly lower than healthy subjects. These findings indicate reduced heart rate variability and lower complexity in MetS group than healthy group. Additional analysis also showed that when subjects had more MetS components, ECG-based heart rate dynamic characters changed significantly in Poincaré SD2 and nonlinear analysis by MSE complexity index, in addition to conventional linear parameters.

Heart rate is intricately regulated by complex interactions of multiple mechanisms, including sympathetic and parasympathetic nervous system, as well as hormonal homeostasis. Cardiac autonomic function is commonly measured non-invasively with HRV, and altered sympathovagal balance can be inferred by both short-term and long-term HRV^29^,^30^. Given the potential mechanism underlying the development of MetS and its major cardiovascular complications, HRV is well recognized for its predictive power. Several HRV parameters have been developed, among them, time and frequency domain measures of HRV were the most commonly used^31^. In a systemic review of 14 studies examining the associations between HRV parameters and MetS, SDNN was the only conventional HRV parameter that was consistently reduced in all 14 studies when one or more risk factors were present compared to zero MetS components^31^. However, in the present study, several conventional parameters (pNN20, LnLF, LnHF) were able to distinguish these populations. Such discrepancy may be due to the heterogeneity in ECG recording duration, study population, accountable variables, body position during ECG recording, and HRV analysis methods among different studies. Methods from nonlinear dynamics have shed new insights into HRV changes under various physiological and pathological conditions, providing additional prognostic information and complementing conventional time and frequency domain analyses. Reduced entropy values have been observed in diabetic patients in comparison with control group^32^,^33^. Khandoker *et al*. further demonstrated that, as compared to conventional HRV indices and Poincaré plot parameters, entropy measure was able to better distinguish diabetic patients with cardiac autonomic neuropathy from the diabetic patient without cardiac autonomic neuropathy^34^. Our present study confirmed the altered cardiac autonomic function in the MetS group with conventional HRV time and frequency domain measures. Complexity index, derived from the MSE analysis, and pNN20 provided the best discrimination between the groups, followed by SDNN, LnLF, LnHF and SD2.

The clustering of various cardiovascular risks referred to as the metabolic syndrome have led to the fact that patients with cardiovascular diseases often have one or more MetS components or undetected diabetes mellitus^9^. Cardiac dynamic alterations are associated with increase cardiovascular risk profile such as insulin-resistance, endothelial dysfunction, arterial stiffening, cardiac hypertrophy, and sympathetic activation^35^. Results from previous studies have shown that metabolic syndrome factors by themselves, or in any combination, portend cardiovascular disease and many other adverse outcomes^36^. The underlying mechanism of MetS and associated cardiac alternation remains unclear. Our previous studies have identified overactivated sympathetic nervous system, as assessed by HRV analysis, in patients with nonalcoholic fatty liver disease (NAFLD), which was also commonly observed in patients with MetS^37^. This association was independent of leptin or subclinical inflammation.

Evidence suggests that both lifestyle and pharmacological interventions can reverse MetS^38^. Metabolic syndrome is conventionally managed by both pharmacological and non-pharmacological approaches^39^, targeting specific core disorders such as obesity, hypertension^40^,^41^,^42^ and hyperlipidaemia. Therefore, since the prevalence of MetS has increased remarkably worldwide, early detection of minute cardiac alternations and early intervention may help to prevent or alleviate the late and more severe cardiovascular complications as a result of MetS in general population. Ambulatory ECG monitoring is easy, accessible, non-invasive, and heart rate based methods are relatively mature techniques for this purpose. In addition, such approach is ideal for the dynamic monitoring of intervention response at multiple times. Conventional or nonlinear methods for heart rate dynamics are feasible as cost-effective approaches for metabolic syndrome. Further interventional studies with exercise or weight loss to modify the severity of metabolic syndrome and/or cardiac autonomic dysfunction may help to elucidate the temporal relationship between the metabolic syndrome and cardiovascular complications.

## Limitations

There are limitations in this study. First, this study was cross-sectional in design, the actual causality between MetS and cardiac autonomic dysfunction could be questioned. In addition, since this study was not specifically designed to investigate cardiovascular damage or cardiac dysfunctions in people with metabolic syndrome, we do not have long-term follow up data yet available. Further longitudinal study is warranted to investigate the impact of heart rate dynamic alterations and long-term health outcomes. Second, we collected at least 4-h ECG recordings during daytime for the present HRV analysis. However, differences in data collection, including the body position, leisure activity, and length of ECG recordings, could affect the HRV analysis and interpretation^31^. Further studies to compare measures during day time wakefulness with the same parameters acquired during sleep, when external influences are minimized, may help to address this important issue. Third, since insulin resistance has been regarded as the underlying pathophysiology of metabolic syndrome, some factors including plasma norepinephrine levels, various adipocytokine levels, fasting insulin, homeostatic model assessment – insulin resistance (HOMA-IR), and unreported medication use were not measured in this study. The possible confounding effects of these factors cannot be totally excluded.

## Conclusion

MetS is significantly associated with alterations in heart rate dynamics. Compared with conventional time and frequency domain HRV measures, Poincaré SD2 analysis and complexity index (derived from MSE) are more sensitive in distinguishing the alterations caused by MetS. Since ambulatory ECG monitoring is readily available and feasible in our clinical practice, large-scale screening to detect early stage cardiac dysfunction may help to prevent or alleviate various late cardiovascular complications.

## Methods

### Materials and Study Design

This prospective study recruited subjects aged equal to or greater than 20 years from a routine health check-ups program in the Health Management Center of National Taiwan University Hospital, starting from January 2012. Attendees of the health check-up in our institute were recruited through advertising messages for health-promotion purposes from the general population and therefore the participants did not belong to any particular socio-economic class or share a unifying form of employment. Subjects with atrial fibrillation, use of ventricular pacing, severe comorbidities, such as congestive heart failure, symptomatic coronary heart disease, uncontrolled pulmonary disease, chronic renal failure, or pregnancy were excluded from the study. Data of medical history, including sleep apnea, was recorded Mental status was evaluated with a validated questionnaire, the five-item Brief Symptom Rating Scale (BSRS-5)^43^ and interviewed by clinicians to approve the eligibility. Information of current use of important medications, including anti-hypertensive agents, hypoglycemic agents and anxiolytics/hypnotics was also comprehensively collected. This study was approved by the ethical committee of National Taiwan University Hospital (No. 201006037R), and we confirm that all experiments were performed in accordance with relevant guidelines and regulations. All subjects have provided written informed consent prior to participating in the study.

The standard protocol of our health check-up program consisted of a self-administered questionnaire, face-to-face interview by an internal medicine physician, physical examination, blood biochemical analysis, and various radiology and gastrointestinal endoscopy exams^44^,^45^. Therefore, analytical data were obtained from this health examination database, with recordings of demography/anthropometry, medical history, medication use, dieting, smoking, alcohol, and level of physical activities. BMI was calculated as weight (kg) divided by height squared (m^2^). Waist circumference was measured at the level of the umbilicus at minimal respiration. Blood pressures were measured at 8 am before taking any medication, and subjects were in the sitting position after sat quietly for 10 min. Systolic and diastolic blood pressures (SBP and DBP) were measured at bilateral upper arms and bilateral thighs, and the reported SBP and DBP in this article were both from upper right arm. Subjects were instructed to fast for at least 10 hours and avoid smoking, alcohol, coffee, and tea on the day of examinations. Comprehensive biometric tests included 110 biomarkers or parameters (i.e., white blood cell count, hemoglobin concentration, fasting blood glucose, high-density lipoprotein cholesterol, triglycerides, uric acid, creatinine, aspartate aminotransferase, and alanine aminotransferase, etc.). The laboratory tests have both internal and external quality control procedures accredited by the Taiwan Society of Laboratory Medicine twice a year.

ECG recordings were collected by an FDA approved ambulatory electrocardiogram monitor (DynaDx Corporation, Taipei, Taiwan) with a computer-based data-acquisition system. The ECG recording equipment was an one-lead Holter device that could record ECG for over 24 hours. All subjects were monitored at home one week after they finished their routine health check-ups to avoid interference. Two long-term ECG recordings were collected during daytime and sleep respectively. All sleep related analysis will be elaborated in another paper^46^. During daytime, all subjects were instructed to wear the device for at least 4 hours and to avoid exercise and naps during recordings. Sampling rate of ECG monitoring was 200 Hz. All ECG recordings were carefully checked with noise level, artifacts, R peak detection and ectopic beats. Data was discarded if less than 4 hours or low quality, or cut if longer than 4 hours.

### Definition of metabolic syndrome

Subjects with MetS were defined by the criteria defined in the Adult Treatment Panel III, with a modification of waist circumference as appropriate for Asians^47^, and was also proposed by the Taiwan National Health Bureau. The five metabolic syndrome characteristic components are: 1) abdominal obesity, defined as WC ≥ 90 cm (in male) or ≥80 cm (in female); 2) elevated blood pressure, measured as SBP ≥ 130 mmHg and/or DBP ≥ 85 mmHg or taking blood pressure-lowering medications; 3) hyperglycemia, fasting blood glucose ≥100 mg/dL (5.6 mmol/L) or taking hypoglycemic medications; 4) hypertriglyceridemia: fasting Triglycerides (TG) ≥150 mg/dL (1.69 mmol/L); and 5) high density lipoprotein (HDL) < 40 mg/dl (in male) or <50 mg/dl (in female). Individuals who were using antidiabetic or antihypertensive therapy were treated as those who met the criteria for high fasting glucose level or high blood pressure. When three of the five listed characteristics were present, a diagnosis of metabolic syndrome was made. Healthy subjects were screened by all past history and were determined as absence of any abnormality of biometric markers, or if they have less than three metabolic syndrome components.

### Heart rate variability (HRV)

Based on non-invasive ECG recordings, heart rate variability (HRV) is a widely used method for assessing activity of the cardiac autonomic nervous system. R-peaks were detected from ECG, and the RR intervals (RRI) were defined as the time intervals between consecutive R peaks. Normal heart beat from the ECG recordings were automatically detected by commercial software (DynaDx Corporation, Taipei, Taiwan) and verified by visual inspections. Ectopic beats were identified and excluded from calculations. Recordings with artifacts or arrhythmias comprising more than 5% of the total epoch were discarded. Thus, normal-to-normal (NN) intervals were extracted for complete HRV analysis by time domain, frequency domain and non-linear analysis. In time domain, mean heart rate (HR), mean of NN intervals (mean NN), standard deviation of NN (SDNN), square root of the mean of the squares of successive N-N interval differences (rMSSD), percentage of heart period differences >20 ms (pNN20) and >50 ms (pNN50) were included. All time domain HRV measurements were analyzed based on the 4-hours ECG recordings. In frequency domain, NN intervals were interpolated and resampled to 4 Hz for HRV frequency domain analysis. The Welch protocol (with a Hamming window applied to each 5 minute segment) was used for spectral analysis. HRV power spectrum measurements were log-transformed to normalize their distribution for analysis. Normalized percentage of LF and HF was defined as nLF = LF/(LF + HF) and nHF = HF/(LF + HF) respectively. Ratio of low frequency over high frequency (LF/HF) was selected to indicate autonomic balance. For non-linear dynamics, Poincaré plot as the two-dimensional reconstructed RR interval phase-spaces was chosen to describe the dynamics of the cardiac system, and multiscale entropy (MSE) was used to analyze the heart rate dynamic complexity.

### Multiscale Entropy (MSE)

MSE analysis was first proposed to evaluate the complexity of physiologic time series, and was well-recognized as a way to assess human health conditions in many studies^26^,^27^,^48^,^49^,^50^,^51^. In human health, decrease of complexity is a common sign of pathological conditions or aging^23^. The MSE applies SampEn (sample entropy) analysis to measure the degree of irregularity of the time series, and SampEn requires the time series being studied to be stationary. Therefore, the retrieved NN intervals were first detrended by Ensemble Empirical Mode Decomposition (EEMD)^52^,^53^,^54^, and the long-term overall trend was removed to improve the stationarity of the time series, and thus the accuracy of entropy calculation. In this study, MSE analysis included 20 scales, and the mean of entropies on all 20 scales was calculated as a complexity index (CI_1–20_).

### MetS Score

Since MetS is characterized by concomitant derangements in multiple factors, MetS Score was proposed by a multiethnic cohort study (6780 subjects)^55^. MetS Score = −11.8769 + (1.5432298 * Log Glucose) + (0.7872732 * Log Triglyceride) − (1.588791 * Log HDL) + (0.0277125 * WC) + (0.0232299 * SBP) + (0.0420722 * DBP) − (0.016408 * Age) − (0.73821 * Gender). For gender in the formula, male = 1 and female = 0. MetS Score is a continuous measure of MetS severity and is proposed as a better predictor of cardiovascular events overall and in individual ethnicities^55^.

### Softwares and Statistical Analyses

MATLAB R2012a (The MathWorks, Inc.) was used for data processing and analysis programming. SPSS 19.0 (IBM SPSS Statistics) was used for statistical analyses. Descriptive statistics were reported as mean ± standard deviation for continuous data, and number (percentage) for categorical data. Comparisons of categorical variables were made using the chi-squared or Fisher’s exact test, where appropriate. Comparisons of continues variables were assessed by t-test or non-parametric test (Mann-Whitney U), where appropriate. Linear and logistic regression models were constructed in sequential models, adjusted for age and gender. A p value < 0.05 was considered statistically significant.

## Additional Information

**How to cite this article:** Ma, Y. *et al*. Cardiac Autonomic Alteration and Metabolic Syndrome: An Ambulatory ECG-based Study in A General Population. *Sci. Rep.* **7**, 44363; doi: 10.1038/srep44363 (2017).

**Publisher's note:** Springer Nature remains neutral with regard to jurisdictional claims in published maps and institutional affiliations.

## Figures and Tables

**Table 1:** Comparison of basic demographics between healthy subjects and subjects with metabolic syndrome.

	MetS (N = 175)	Healthy (N = 226)	p	p^†^
Demographic				
Age (yr)	54.76 ± 9.04	49.96 ± 8.86	<0.001**	
Gender (F/M)	52/123	111/115	<0.001**	
Body Composition				
BMI (kg/m^2^)	26.94 ± 3.53	22.88 ± 2.9	<0.001**	<0.001**
WC (cm)	93.93 ± 8.11	82.9 ± 8.41	<0.001**	<0.001**
Body fat (%)	29.51 ± 5.09	25.51 ± 5.44	<0.001**	<0.001**
Exam Parameters				
SBP (mmHg)	129.78 ± 15.36	113.37 ± 12.35	<0.001**	<0.001**
DBP (mmHg)	78.07 ± 10.22	68.08 ± 8.95	<0.001**	<0.001**
T-CHO (mg/dL)	196.34 ± 36.71	197.65 ± 34.26	0.713	0.634
TG(mg/dL)	189.1 ± 112.08	97.63 ± 38.94	<0.001**	<0.001**
HDL (mg/dL)	40.51 ± 8.01	54.35 ± 13.34	<0.001**	<0.001**
LDL (mg/dL)	123.93 ± 30.58	123.88 ± 29.84	0.988	0.643
TP (g/L)	7.31 ± 0.37	7.32 ± 0.41	0.728	0.936
FBG (mg/dL)	105.86 ± 20.71	90.33 ± 7.83	<0.001**	<0.001**
GLU 2-hr PC(mg/dL)	139.99 ± 51.74	109.5 ± 30.86	<0.001**	<0.001**
HbA1c (%)	6.01 ± 0.84	5.5 ± 0.3	<0.001**	<0.001**
hs-CRP (mg/L)	0.22 ± 0.32	0.15 ± 0.29	0.036*	0.031*
Hb (g/dL)	14.91 ± 1.46	14.3 ± 1.47	<0.001**	0.039*
UA (mg/dL)	6.34 ± 1.52	5.48 ± 1.32	<0.001**	<0.001**
Drinking			0.890	0.683
No drinking	61 (35.9%)	76 (35.3%)		
Occasional drinking	86 (50.6%)	113 (52.6%)		
Alcohol intake	23 (13.5%)	26 (12.1%)		
Smoking			<0.001**	<0.001**
Never smokers	107 (61.1%)	183 (81.0%)		
Former Smokers	40 (22.9%)	26 (11.5%)		
Current smokers	28 (16.0%)	17 (7.5%)		
Current Medications				
anti-hypertensive agents	72 (41.1%)	0	<0.001**	0.996
hypoglycemic agents	30 (17.1%)	0	<0.001**	0.998
anxiolytics/hypnotics	12 (6.9%)	18 (8.0%)	0.707	0.365
Mental State Evaluation				
BSRS-5 score	2.52 ± 3.10	2.95 ± 3.38	0.190	0.968
Sleep Apnea	27 (15.4%)	9 (4.0%)	<0.001**	0.005**

BMI, body mass index; WC, waist circumference; SBP, systolic blood pressure; DBP, diastolic blood pressure; T-CHO, total cholesterol; TG, triglycerides; HDL, high density lipoprotein; LDL, low density lipoprotein; TP, total protein; FBG, Fasting blood glucose; GLU 2-hr PC, 2-hr Postprandial glucose; HbA1c, Hemoglobin A1c; hs-CRP, high-sensitivity C-reactive protein; Hb, hemoglobin; UA, Uric acid; BSRS-5, five-item Brief Symptom Rating Scale.

Alcohol intake was defined as drinking at least once a week. Occasional drinking was defined as drinking less than once a week.

Values reported are either number (percentages) or mean ± standard deviation. *p < 0.05; **p < 0.01.

p^†^ indicates p value adjusted for age and gender.

**Table 2:** Comparison of daytime characteristics of various ECG-based parameters between healthy subjects and subjects with metabolic syndrome.

	MetS (N = 175)	Healthy (N = 226)	p	p^†^	p^#^
Time Domain					
Mean HR	83.41 ± 12.05	82.38 ± 11.42	0.383	0.013*	0.010*
Mean NN (ms)	741.03 ± 105.68	750.71 ± 105.53	0.363	0.014*	0.016*
SDNN	70.82 ± 19.90	76.93 ± 17.59	0.001**	0.001**	0.008*
rMSSD (ms)	20.55 ± 14.30	21.79 ± 10.23	0.314	0.497	0.967
pNN20 (%)	17.09 ± 14.41	21.44 ± 12.94	0.002**	0.026*	0.273
pNN50 (%)	3.85 ± 8.40	3.94 ± 6.27	0.895	0.801	0.431
Frequency Domain					
LnTP	14.97 ± 0.54	15.01 ± 0.41	0.407	0.066	0.038
LnVLF	9.40 ± 0.70	9.62 ± 0.60	0.001**	0.004**	0.057
LnLF	8.09 ± 0.81	8.50 ± 0.73	<0.001**	<0.001**	0.014*
LnHF	6.81 ± 0.91	7.18 ± 0.84	<0.001**	0.001**	0.046*
nLF (%)	76.66 ± 12.00	77.28 ± 10.82	0.587	0.674	0.853
nHF (%)	23.34 ± 12.00	22.72 ± 10.82	0.587	0.674	0.853
LF/HF (nu)	4.31 ± 2.43	4.35 ± 2.37	0.871	0.787	0.945
Poincaré plot					
SD1 (ms)	14.54 ± 10.12	15.41 ± 7.24	0.315	0.496	0.968
SD2 (ms)	98.57 ± 28.18	107.44 ± 24.94	0.001**	0.001**	0.006**
SD1/SD2	0.16 ± 0.12	0.15 ± 0.08	0.477	0.406	0.187
Multiscale Entropy					
Complexity index (CI_1–20_)	1.69 ± 0.18	1.77 ± 0.12	<0.001**	<0.001***	0.005**

NN, time interval between each normal heart beat; SDNN, the standard deviation of NN; rMSSD, square root of the mean of the squares of successive NN interval differences; pNN20, percentage of heart period differences >20 ms; pNN50, percentage of heart period differences >50 ms; LnTP, log form of total power; LnVLF, log form of very low frequency power; LnLF, log form of low frequency power; LnHF, log form of high frequency power.

SD1, normalized deviation of instantaneous beat-to-beat N-N interval variability in the short diameter; SD2, normalized deviation of instantaneous beat-to-beat NN interval variability in the long diameter; LF/HF, ratio of low frequency over high frequency.

Values reported are mean ± standard deviation. *p < 0.05; **p < 0.01.

p^†^ indicates p value adjusted for age and gender; p^#^ indicates p value adjusted for all confounding variables, including age, gender, medications, and diagnosis of sleep apnea.
